# Supplementary material for: Persistent and Dose-Dependent Neural and Metabolic Gene Expression Changes Induced by Transient Citalopram Exposure in Zebrafish Embryos
Source: Int J Mol Sci. 2025 Nov 22;26(23):11288. doi: 10.3390/ijms262311288 (PMC12692716; doi:10.3390/ijms262311288)
Supplement: Supplementary file 1 [file ijms-26-11288-s001.zip › Supplemental Figures_Citalopram.pdf]

A

	Gene Set	Description	Size	Expect	Ratio	P Value	FDR
**KEGG**	dre03010	Ribosome	128	1.504	4.6544	0.000721	0.12397
	dre04510	Focal adhesion	258	3.0314	2.639	0.010514	0.90423
**GO:BP**	GO:0009792	embryo development ending in birth or egg hatching	461	4.4649	3.1356	0.000147	0.058035
	GO:0035148	tube formation	61	0.5908	8.4631	0.000303	0.059859
**GO:CC**	GO:0005840	ribosome	174	2.0474	3.419	0.004214	0.44667
	GO:0045182	translation regulator activity	133	1.3015	3.8416	0.009768	0.78832

B

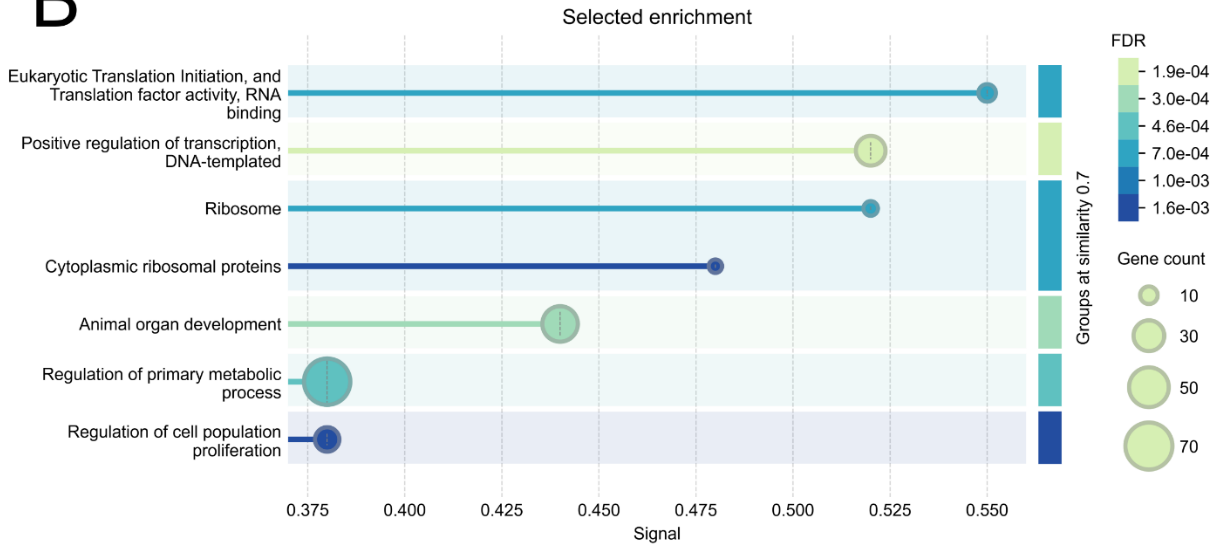

Supplemental Figure S1. Over-representation analysis (ORA) of control versus 0.03 ug/L citalopram. (**A**) Table of significantly enriched KEGG pathways and GO biological processes (GO:BP) and cellular component (GO:CC) terms for control versus 0.03 ug/L citalopram, showing gene set size, expected count, enrichment ratio, raw p-value, and false discovery rate (FDR). (**B**) Enrichment map of selected functional categories of control vs 0.03 ug/L citalopram with bubble size representing gene count and color scale indicating FDR significance. “Signal” is a composite enrichment score calculated from the proportion of input genes overlapping with the category and the statistical significance of that overlap (p-value).

		Description	LEN/Size	NES	P Value	FDR
**0.03 ug/L**	**KEGG**	Citrate cycle (TCA cycle)	21/32	1.7998	0.001075	0.026265
		Adherens junction	61/134	1.8223	<2.2e-16	0.027053
		Glyoxylate and dicarboxylate metabolism	22/35	1.7646	0.002108	0.032711
		Glycine, serine and threonine metabolism	5/11	1.8384	0.000518	0.040734
		Oxidative phosphorylation	59/134	1.7141	<2.2e-16	0.045226
	**GO:BP**	regulation of membrane potential	31/118	-2.0379	<2.2e-16	0.016032
		ectodermal placode development	8/17	1.8608	0.000791	0.046637
	**GO:CC**	collagen trimer	23/48	2.0352	<2.2e-16	0.000323
		oxidoreductase complex	5/9	1.9166	<2.2e-16	0.001375
		transcription regulator complex	91/213	1.7018	<2.2e-16	0.030462
		synaptic membrane	13/50	-1.8188	<2.2e-16	0.032301
		neuron projection terminus	5/27	-1.7322	0.005181	0.040691
	**GO:MF**	structural constituent of eye lens	23/39	2.2944	<2.2e-16	<2.2e-16
		transcription factor binding	33/67	2.0839	<2.2e-16	9.6E-05
		glutamate receptor activity	5/14	-2.288	<2.2e-16	0.000941
		neurotransmitter receptor activity	19/67	-2.0936	<2.2e-16	0.003648
		amide transmembrane transporter activity	16/51	-1.8694	0.000943	0.019141
**0.9 ug/L**	**GO:CC**	axon	21/53	-1.86	<2.2e-16	0.01972
		neuron projection terminus	4/9	-1.875	0.003674	0.023843
		postsynapse	29/74	-1.9098	<2.2e-16	0.029638
		somatodendritic compartment	21/71	-1.679	0.000815	0.043755
		synaptic membrane	73/149	-1.9536	<2.2e-16	0.043959
	**GO:MF**	structural constituent of eye lens	20/39	2.1753	<2.2e-16	0.000203
		organophosphate ester transmembrane transporter activity	12/31	2.0031	<2.2e-16	0.003544
		glutamate receptor activity	14/27	-2.1208	0.001194	0.034964
**50 ug/L**	**KEGG**	Glutathione metabolism	9/19	2.0861	<2.2e-16	0.001017
		Arachidonic acid metabolism	21/44	2.0922	<2.2e-16	0.001743
		Ferroptosis	7/13	2.0136	<2.2e-16	0.00184
		Glycerolipid metabolism	4/9	1.9851	<2.2e-16	0.002179
		Cardiac muscle contraction	41/126	-2.0021	<2.2e-16	0.004976
	**GO:BP**	synaptic signaling	119/250	-2.7878	<2.2e-16	<2.2e-16
		skin development	20/33	2.2042	<2.2e-16	0.000127
		response to toxic substance	32/69	2.1193	<2.2e-16	0.000254
		monoamine transport	2/3	-2.1169	<2.2e-16	0.00032
		regulation of membrane potential	69/118	-2.1246	<2.2e-16	0.000333
	**GO:CC**	transporter complex	51/125	-2.247	<2.2e-16	<2.2e-16
		presynapse	11/34	-2.3678	<2.2e-16	<2.2e-16
		somatodendritic compartment	21/53	-2.416	<2.2e-16	<2.2e-16
		postsynapse	95/223	-2.5259	<2.2e-16	<2.2e-16
		axon	107/210	-2.7147	<2.2e-16	<2.2e-16
		mitochondrial envelope	169/417	1.5969	<2.2e-16	0.048578
	**GO:MF**	cysteine-type endopeptidase regulator activity involved in apoptotic process	8/11	2.325	<2.2e-16	<2.2e-16
		glutamate receptor activity	19/28	-2.4584	<2.2e-16	<2.2e-16
		oxidoreductase activity, acting on peroxide as acceptor	19/35	2.2696	<2.2e-16	7.99E-05
		structural constituent of eye lens	34/39	-2.0964	0.000447	0.00081
		passive transmembrane transporter activity	118/341	-2.0249	<2.2e-16	0.001367
**250 ug/L**	**GO:BP**	synaptic signaling	107/253	-2.145	<2.2e-16	0.001288
		regulation of membrane potential	45/118	-2.0343	<2.2e-16	0.005081
		glutamate receptor signaling pathway	17/31	-2.0031	0.000488	0.005439
		monoamine transport	11/24	-1.8789	0.000948	0.016389
		muscle system process	33/94	-1.8406	<2.2e-16	0.019037
	**GO:CC**	synaptic membrane	67/151	-2.0285	<2.2e-16	0.002246
		transporter complex	83/251	-1.9714	<2.2e-16	0.00347
		cell body	44/91	-1.9283	<2.2e-16	0.004015
		axon	23/70	-1.8903	<2.2e-16	0.004644
		somatodendritic compartment	7/18	-1.849	<2.2e-16	0.005838
	**GO:MF**	neurotransmitter receptor activity	37/69	-2.2848	<2.2e-16	0.000226
		glutamate receptor activity	17/28	-2.1517	<2.2e-16	0.000677

Supplemental Table S1. Top pathway terms per category resulting from gene set enrichment analysis (GSEA) according to dose of citalopram (0.03, 0.9, 50, and 250 μg/L). Pathway categories include KEGG, GO: Biological process (BP), Cellular component (CC), and Molecular function (MF). For each term there is an associated leading-edge number (LEN), size of term category (Size), normalized enrichment score (NES), p-value, and false discovery rate (FDR).

	**0.03 ug/L**		**0.9 ug/L**		**50 ug/L**		**250 ug/L**	
	**padj**	**arcsin sqrt pvalue**	**padj**	**arcsin sqrt pvalue**	**padj**	**arcsin sqrt pvalue**	**padj**	**arcsin sqrt pvalue**
**Basal2a**	0.2797	0.0524	0.2797	0.0555	0.3466	0.2046	0.3111	0.1235
**MusSkelFast2a**	0.3380	0.2055	0.4397	0.2675	0.2797	0.0568	0.4397	0.0883
**Neutrophil**	0.2797	0.0398	0.3111	0.1214	0.2797	0.0797	0.3380	0.1596
**MucNCC**	0.2797	0.0905	0.3466	0.2206	0.2797	0.0741	0.3466	0.2294
**MHBNeur25**	0.2797	0.0486	0.3998	0.2822	0.2918	0.1073	0.4451	0.4321
**LensAll**	0.2797	0.0314	0.4316	0.3491	0.4204	0.3086	0.4204	0.3106
**Ncirid**	0.4316	0.3306	0.3466	0.2006	0.4204	0.3153	0.2797	0.0354
**RetDiff25c**	0.2797	0.0889	0.2918	0.1045	0.3380	0.1640	0.3466	0.1870
**FinBud2**	0.2797	0.0487	0.3380	0.1591	0.3380	0.1562	0.4316	0.3450
**HairCell**	0.3380	0.1462	0.2797	0.0493	0.3466	0.2289	0.3466	0.1870
**MHBNeurGlutAll**	0.2797	0.0513	0.4451	0.4233	0.2797	0.0855	0.3466	0.2141
**IonovH**	0.3466	0.2218	0.4389	0.3724	0.2797	0.0622	0.4451	0.4281

Supplemental Table S2. Single-cell deconvolution analysis of transcriptomic changes across citalopram exposure concentrations. Cell type proportions were estimated for each exposure group using single-cell deconvolution. The table shows adjusted p-values (padj) and arcsine root-transformed p-values for each cell type at 0.03, 0.9, 50, and 250 ug/L citalopram. No cell type reached statistical significance after multiple testing corrections, but nominal p-values suggest potential shifts in neuronal, lens, and immune cell populations.
