# Supplementary figures and images for: Persistent and Dose-Dependent Neural and Metabolic Gene Expression Changes Induced by Transient Citalopram Exposure in Zebrafish Embryos
Source: Int J Mol Sci. 2025 Nov 22;26(23):11288. doi: 10.3390/ijms262311288 (PMC12692716; doi:10.3390/ijms262311288)

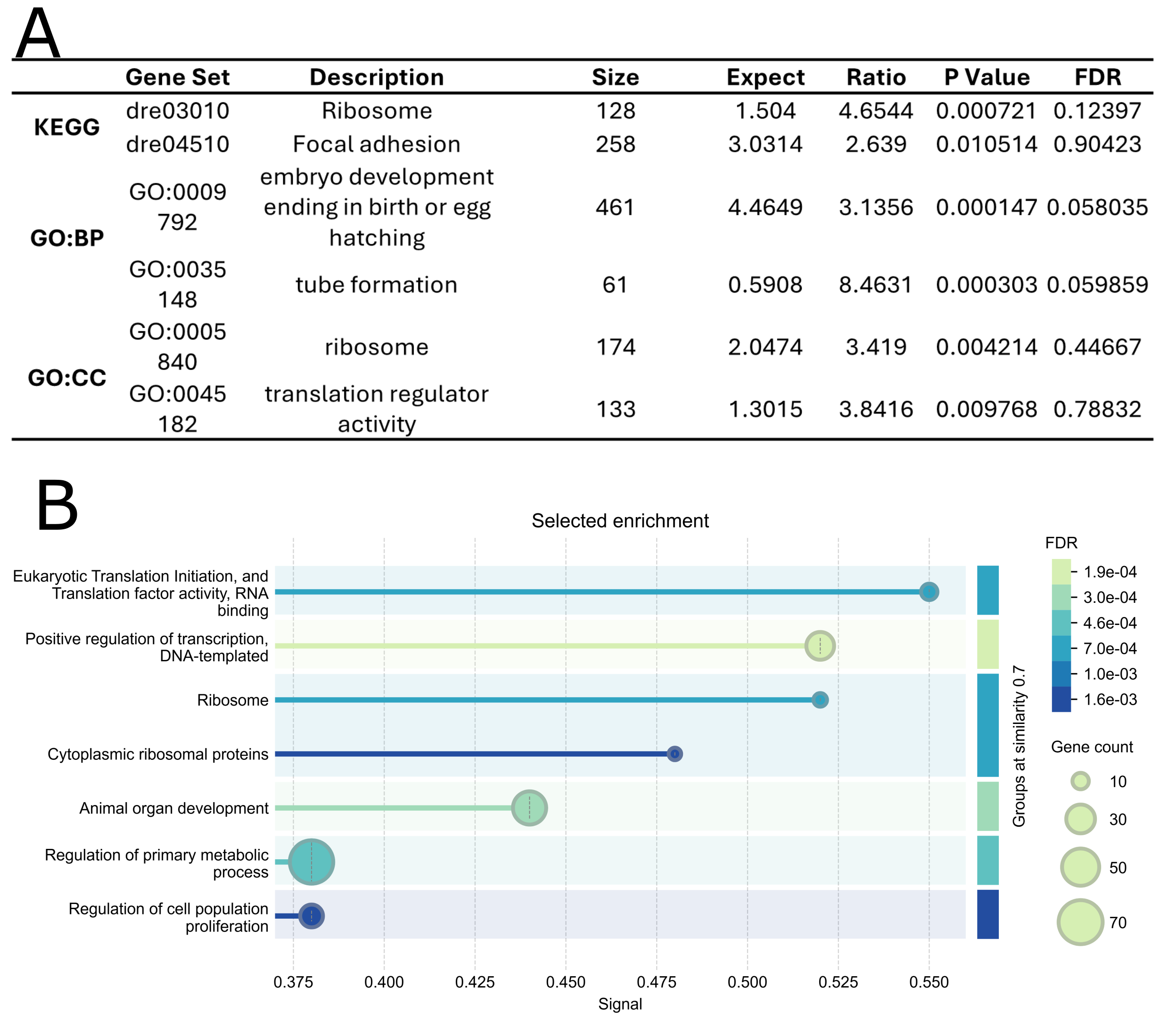

Supplement: Supplementary file 1 [file ijms-26-11288-s001.zip › Supplemental_Figure_S1.png]

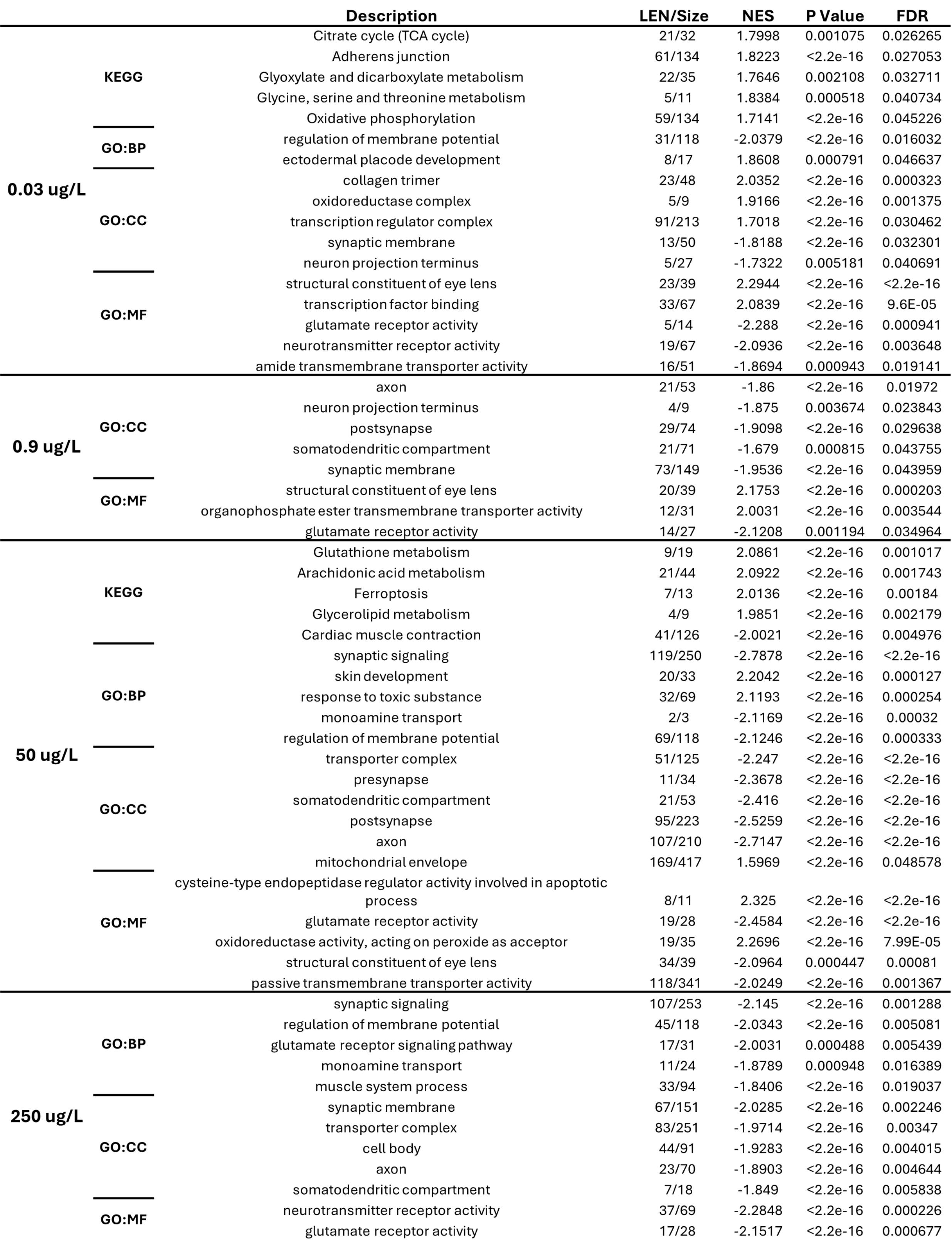

Supplement: Supplementary file 1 [file ijms-26-11288-s001.zip › Supplemental_Table_S1.tiff]

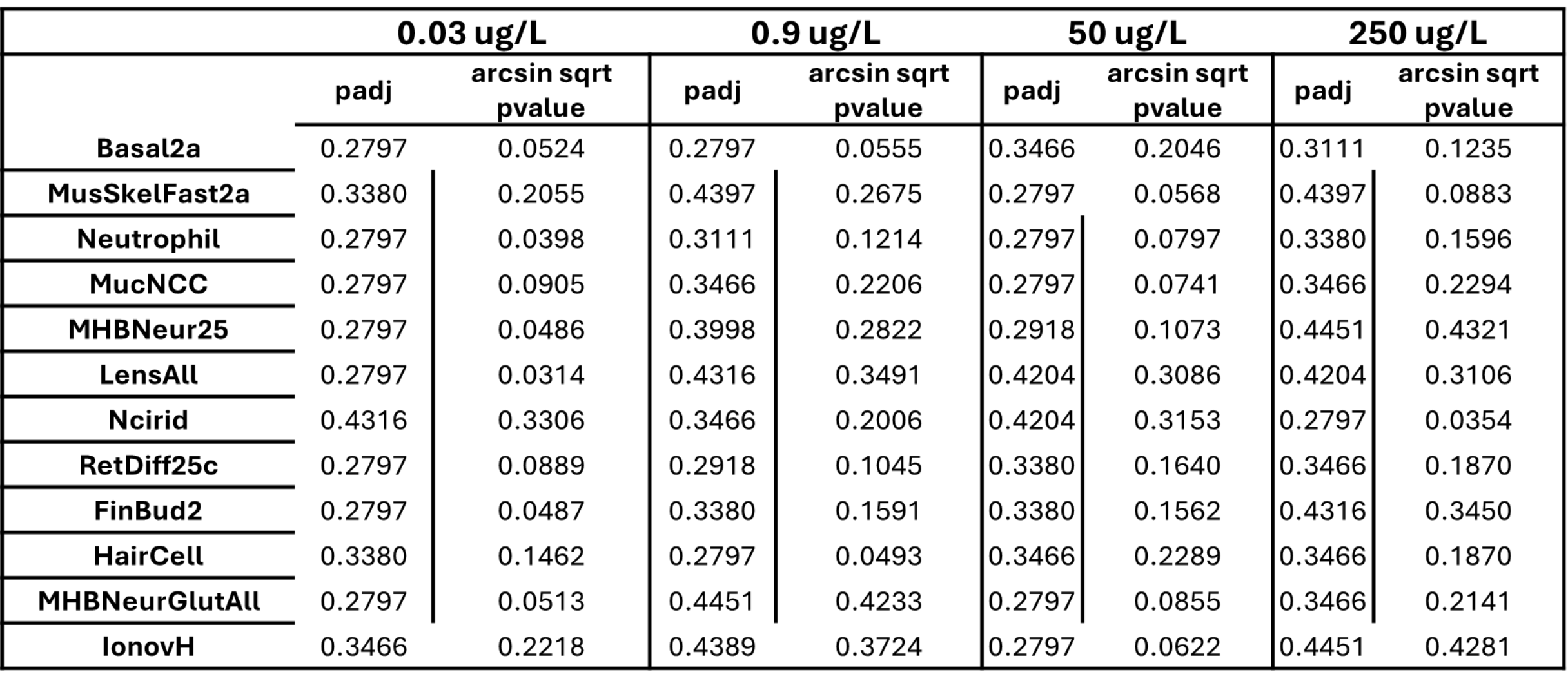

Supplement: Supplementary file 1 [file ijms-26-11288-s001.zip › Supplemental_Table_S2.png]
